# Genome-Wide Association Studies Identify Candidate Genes for Coat Color and Mohair Traits in the Iranian Markhoz Goat

**DOI:** 10.3389/fgene.2018.00105

**Published:** 2018-04-04

**Authors:** Anahit Nazari-Ghadikolaei, Hassan Mehrabani-Yeganeh, Seyed R. Miarei-Aashtiani, Elizabeth A. Staiger, Amir Rashidi, Heather J. Huson

**Affiliations:** ^1^Department of Animal Science, College of Agriculture and Natural Resources, University of Tehran, Karaj, Iran; ^2^Department of Animal Science, Cornell University, Ithaca, NY, United States; ^3^Department of Animal Science, Faculty of Agriculture Engineering, University of Kurdistan, Sanandaj, Iran

**Keywords:** genome-wide association study, coat color, mohair, fleece, Markhoz, goat, Angora, wattles

## Abstract

The Markhoz goat provides an opportunity to study the genetics underlying coat color and mohair traits of an Angora type goat using genome-wide association studies (GWAS). This indigenous Iranian breed is valued for its quality mohair used in ceremonial garments and has the distinction of exhibiting an array of coat colors including black, brown, and white. Here, we performed 16 GWAS for different fleece (mohair) traits and coat color in 228 Markhoz goats sampled from the Markhoz Goat Research Station in Sanandaj, Kurdistan province, located in western Iran using the Illumina Caprine 50K beadchip. The Efficient Mixed Model Linear analysis was used to identify genomic regions with potential candidate genes contributing to coat color and mohair characteristics while correcting for population structure. Significant associations to coat color were found within or near the *ASIP, ITCH, AHCY*, and *RALY* genes on chromosome 13 for black and brown coat color and the *KIT* and *PDGFRA* genes on chromosome 6 for white coat color. Individual mohair traits were analyzed for genetic association along with principal components that allowed for a broader perspective of combined traits reflecting overall mohair quality and volume. A multitude of markers demonstrated significant association to mohair traits highlighting potential candidate genes of *POU1F1* on chromosome 1 for mohair quality, *MREG* on chromosome 2 for mohair volume, *DUOX1* on chromosome 10 for yearling fleece weight, and *ADGRV1* on chromosome 7 for grease percentage. Variation in allele frequencies and haplotypes were identified for coat color and differentiated common markers associated with both brown and black coat color. This demonstrates the potential for genetic markers to be used in future breeding programs to improve selection for coat color and mohair traits. Putative candidate genes, both novel and previously identified in other species or breeds, require further investigation to confirm phenotypic causality and potential epistatic relationships.

## Introduction

Throughout history, goats have played a vital role in the livelihood of humans, being a main source of meat, milk, fiber, and hides, especially in harsh environmental conditions. Archeological evidence supports goat domestication around 10,000 years ago in the Zagros Mountain region in Iran (Zeder and Hesse, 2000) with the UN Food and Agriculture Organization (FAO) estimating 25 million goats in the country today (FAOSTAT, 2008). Many goat breeds, including the Iranian Markhoz breed, have adapted to climates with extremely high temperatures, low rainfall, and low humidity. Yet it is the mohair or long, silky hair of the Markhoz goat that make this indigenous breed unique. Mohair is generally associated with the popular Angora breed and as such, the Markhoz goat is oftentimes considered an Angora goat. The Markhoz goat is the only mohair-producing breed within the Kurdistan province in the west of Iran. A prominent feature of this breed is the coat color variation, which can be dark to light brown, black, gray, or white. This coat color variation is unique among Angora goats which are predominantly selected for a white coat color (Rashidi et al., 2006). The mohair, particularly the brown color from the Markhoz goats, is often used to make clothing for important cultural ceremonies, especially weddings. Unfortunately, due to a reduction in population size, a genetic bottleneck has been observed in the Markhoz population reducing diversity (Rashidi et al., 2015). Cross breeding and inbreeding are additional concerns for all local breeds as inbreeding is likely increasing as population sizes diminish and innate characteristics of the indigenous breeds may be lost during admixture with other breeds (Hanotte et al., 2010).

While the Markhoz goat is indigenous to Iran, Angora goats are now found predominantly in South Africa, the United States, and Argentina, with smaller herds in other countries like Turkey, Australia, and New Zealand (Mohair South Africa, 2017). Mohair production is unique to these animals in that they are the only single coated breeds in which the primary and secondary hair follicles produce the same fiber (Mohair South Africa, 2017). Adult Angora goats are typically shorn twice a year in the aforementioned major production countries providing 2–2.5 kg of mohair per goat (Mohair South Africa, 2017). Mohair is a type of fiber collected from Markhoz and Angora goats that is exceptionally soft, has a high luster, and is used worldwide in the textile and clothing industries. The softness of mohair and other quality traits are determined based on the hair’s diameter, kemp, and medullated and greasy fiber content. Kemp is an undesirable fiber characteristic that causes irregular dying properties and a coarse appearance due to the medullated hair fibers having a core of air-filled cells and course medulla. The less kemp, the better the mohair quality and true fiber percentage of the fleece. Increased greasy fiber percentage or kemp percentage is unfavorable for industry purposes, yet both qualities are important adaptive mechanisms which protect the mohair against humidity and environmental contamination such as dirt and vegetation (Mohair South Africa, 2017). A softer hair fiber has a smaller diameter with less kemp, and less medullated and greasy fiber content. True fiber is considered pure fiber without kemp while fiber efficiency equates clean fleece without any grease content.

There are few genome-wide studies for production and disease related traits in goats as compared to other mammals (Zidi et al., 2014; Becker et al., 2015; Lan et al., 2015; Reber et al., 2015; Martin P. et al., 2016; Martin P.M. et al., 2016; Menzi et al., 2016). To date, goats are not included in a Quantitative Trait Loci (QTL) database (Hu et al., 2016). However, linkage analysis has identified QTL regions for coefficient of variation of fiber diameter, kemp fiber, discontinuous medullated fiber, staple length, fleece weight, fiber diameter, and comfort factor spinning fineness in Angora goats and for fleece yield in Cashmere goats (Cano et al., 2007, 2009; Visser et al., 2011; Roldan et al., 2014). The keratin (*KRT*) and keratin associated protein (*KRTAP*) family genes located on chromosomes 1 and 5 were highlighted as candidate genes potentially responsible for diameter and kemp traits in Angora goats and have been previously confirmed in sheep (Parsons et al., 1994; Cano et al., 2007). More recently, whole-genome sequences of two Chinese breeds of cashmere goats were compared for signatures of selection and identified genes and biological pathways potentially related to cashmere production (Li et al., 2017). Gene editing of the fibroblast growth factor 5 (*FGF5*) gene in goat embryos resulted in an increased number of second hair follicles and longer fiber length which would suggest greater cashmere production (Wang et al., 2016).

The vast majority of Markhoz goats have a brown coat color likely due to the higher value of this color fiber for cultural events. Coat color pigmentation is a polygenic trait with genes often having epistatic interactions (Sturm et al., 2001). Well-known genes involved in coat color include melanocyte-stimulating hormone receptor (*MC1R*) and agouti signaling protein (*ASIP* or *Agouti*) which have a consistent effect across many species. The *MC1R* gene plays a key role in melanin color theme synthesis and the concentration of eumelanin or pheomelanin, which can lead to the black/brown or red/yellow phenotype, respectively. There are several studies that have investigated *MC1R* in cattle and sheep for coat color patterns (Klungland et al., 1995; Joerg et al., 1996; Våge et al., 1999; Fontanesi et al., 2009a; Switonski et al., 2013). Similarly, mutations in the *MC1R* gene have been associated with various coat color patterns in the Girgentana, Maltese, Derivata di Siria, Murciano-Granadina, Camosciata delle Alpi and Saanen goats as well (Fontanesi et al., 2009a). *ASIP* has an epistatic effect with *MC1R* and can reduce *MC1R* activity to generate more pheomelanin by preventing cAMP production. Yellow or pheomelanin pigmentation is a result of a dominant allele (*A*) at the *ASIP* locus, while recessive allele (*a*) produces eumelanin resulting in the black/brown phenotype (Adalsteinsson et al., 1994). In Saanen goats, the dominant A^Wt^ (white/tan) allele seems to be responsible for the white coat color (Martin P.M. et al., 2016). In sheep, a gene duplication in the *ASIP* gene is responsible for white and black phenotypes (Norris and Whan, 2008). Another gene for coat color is proto-oncogene receptor tyrosine kinase (*KIT*) which plays a key role for different white color patterns in the pig, cat, cow, mouse, horse, rabbit, dog, and camel (Geissler et al., 1988; Marklund et al., 1998; Pielberg et al., 2002; Haase et al., 2009, 2015; Fontanesi et al., 2010a,b, 2014; Wong et al., 2013; David et al., 2014; Durig et al., 2017; Holl et al., 2017).

The aim of this study was to characterize mohair and coat color phenotypes in Markhoz goats, and identify candidate genes likely influencing these characteristics by exploring the entire genome. Traits included true fiber percentage, grease percentage, kemp percentage, efficiency, diameter, staple length, mature fleece weight and yearling fleece weight. Additionally, we performed principal component analysis (PCA) of the mohair traits to combine qualities into a single variable. The use of PCA generated two new traits with PC1 reflecting fiber quality and PC2 reflecting fiber volume. Candidate genes were identified through genome-wide association of genetically characterized goats using the Illumina Caprine 50K beadchip (Tosser-Klopp et al., 2014). Genome-wide association studies (GWAS) were conducted with significant associations identifying putative candidate genes including *ASIP* and *KIT* genes for brown/black and white coat color, respectively. These studies support the established roles of well-known genes such as *ASIP* and *KIT* in coat color, and identify novel genes such as melanoregulin (*MREG*), which potentially influence mohair quality. Additionally, specific genetic markers and haplotypes are identified which show potential for use in genetic selection schemes for coat color. This provides a foundation to further investigate the biological pathways and causative mutations influencing industry-valued qualities of mohair and the biological implication to animal adaptation.

## Materials and Methods

### Animals and Phenotypes

**FIGURE 1:**
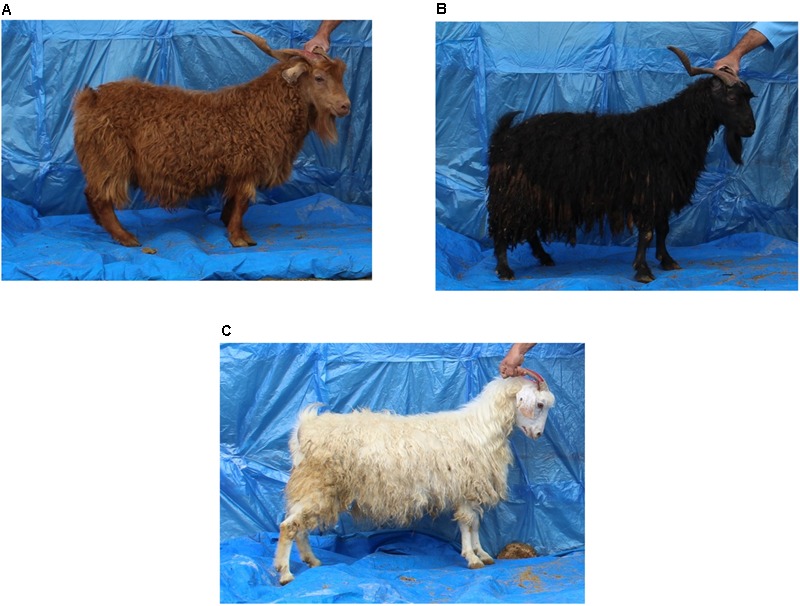
Coat color variation in Markhoz goats used for a genome-wide association study. **(A)** Brown (*n* = 168), **(B)** black (*n* = 26), **(C)** white (*n* = 26).

Coat color and seven fleece traits from a total of 228 Markhoz goats (44 males and 184 females) were sampled at the Markhoz goat Research Station in Sanandaj, Kurdistan province, located in western Iran. All animal procedures were approved by the Cornell University Institutional Animal Care and Use Committee prior to sampling (protocol #2014-0121), and were conducted in a manner to minimize animal stress and handling. Goats ranged in age from 1 to 7 years old. Fleece traits included diameter, kemp percentage, staple length, true fiber percentage, efficiency, grease percentage, and fleece weight. True fiber is considered pure fiber without kemp. Fiber efficiency is equivalent to the clean fleece with no grease and can be calculated by subtracting clean fleece weight from greasy fleece weight and dividing this total by the greasy fleece weight. Coat color was classified into three different categories: brown (*n* = 168), black (*n* = 26), or white (*n* = 26; **Figure 1**). Coat colors have been routinely recorded by the research station since 1992. Photographs were taken of each animal at sample collection according to the USDA_AGIN Goat Sample Protocol (USDA, 2016).

### Statistical Analysis

All animals were assessed for data quality and completeness. Statistical analysis was performed using proc GLM in SAS studio university edition (SAS Institute Inc., Cary, NC, United States) for each fleece trait to determine their relationship to variables such as sex, age (from 1 to 7 years), dam’s age (2 to 8 years), type of birth (single, twin, or triplet) and color (black, brown, or white) for subsequent inclusion as covariates in the GWAS. The specific number of animals used in each GWAS is denoted in **Table 1** and varies based on the number of animals with quality trait information and the GWAS model chosen. We also performed least square means and used the Tukey method for comparing means of males and females for yearling fleece weight and comparing coat colors (black, brown, and white) for fiber volume. PCA was performed on seven of the fiber traits for animals with complete records across all fiber traits using JMP PRO 12 (SAS Institute Inc., Cary, NC, United States). The correlation matrix was applied due to the wide variation in quantitative measures of each trait. PCA was used to generate single quantitative variables combining the seven mohair traits with principal components retained for interpretation and analysis if the eigenvalue score was greater than 1.0.

**Table 1:** Genome-wide association studies conducted for coat color and mohair traits in Markhoz goats.

Trait	Model	# Individuals	Covariates
Brown coat color	Case-Control/Additive	168 Brown, 52 controls	–
Black coat color	Case-Control/Additive	26 Black, 194 controls	–
White coat color	Case-Control/Additive	26 White, 194 controls	–
Brown vs. Black	Case-Control/Additive	168 Brown, 26 Black	–
Black vs. White	Case-Control/Additive	26 Black, 26 White	–
Brown vs. White	Case-Control/Additive	168 Brown, 26 White	–
PC1 Mohair traits: “Fiber quality”	Case-Control/Dominant	29 cases, 76 controls	Sex
PC2 Mohair traits: “Fiber volume”	Categorical/Dominant	105 individuals in 4 categories	Sex, color
Diameter	Case-Control/Dominant	69 cases, 69 controls	Sex, age, color
True fiber percentage	Quantitative/Additive	138 individuals	Sex, color
Kemp percentage	Case-Control/Recessive	21 cases, 94 controls	–
Grease percentage	Quantitative/Additive	138 individuals	–
Efficiency	Case-Control/Dominant	69 cases, 69 controls	Sex
Staple length	Case-Control/Recessive	34 cases, 96 controls	Age, color
Fleece weight	Case-Control/Recessive	14 cases, 114 controls	Age
Normalized Yearling fleece weight	Case-Control/Dominant	34 cases, 161 controls	Sex, year

*The parameters for each trait association including the model of best fit, number of individuals assessed, and covariates used are stated.*

### Genotyping and Quality Control

Whole blood (5 ml) was obtained via the jugular vein into vacutainers with the anticoagulant K_2_EDTA for subsequent DNA extraction. Genomic DNA was extracted following a standard Phenol-Chloroform extraction protocol (Sambrook and Russell, 2006). All samples were genotyped on the Illumina Caprine 50K beadchip (Illumina, Inc., San Diego, CA, United States) at VHL Genetics (VHL Genetics, Wageningen, Netherlands). The initial 53,347 SNPs were assessed for quality and removed if they had a call rate less than 0.9 (*n* = 624) and a minor allele frequency less than 0.03 (*n* = 2540). An additional 419 SNPs, unassigned to a chromosome position, were removed, leaving 49,764 SNPs for analysis. Five samples were subsequently removed with a genotyping call rate less than 0.9. To evaluate population structure and relatedness, we used an identity-by-state (IBS) similarity matrix to calculate genome-wide identity-by-descent (IBD) estimates. Three animals were removed due to having an estimated IBS score greater than 0.90 denoting substantial relatedness. Genotype quality control was conducted using Golden Helix SVS v8.3.4 (Golden Helix, Bozeman, MT, United States).

### Genome-Wide Association Studies

Multiple genome-wide tests were performed for coat color and mohair traits (**Table 1**) using Golden Helix SVS v8.3.4 (Golden Helix, Bozeman, MT, United States). Two hundred and twenty individuals were included in the coat color GWAS and 138 individuals were included in the mohair trait GWAS including 179 females and 41 males, and 115 females and 23 males, respectively. Quantitative or case-control associations were used in an Efficient Mixed Model Linear analysis (EMMAX) (Kang et al., 2010) to correct for remaining population structure and relatedness by including genomic relationship matrix as a random effect in a model. Coat color GWAS were performed in a case-control study design comparing the identified coat color to all other coat colors combined, including brown compared to black and white, black compared to brown and white and white compared to black and brown. Additional GWAS were evaluated with smaller sample sizes to compare single coat colors to one another (i.e., brown compared to black). Variation in brown coat color was considered but small sample size and insufficient differentiation between color variations precluded an association analysis. Association studies with the covariates of sex (male or female), age (1–7 years old), dam’s age (2–8 years old), and type of birth (single or twins) were considered in additive, dominant, and recessive inheritance models. Quantile–quantile (QQ) plots were used to determine the model of best fit for each trait. Quantitative measures were used in the GWAS for true fiber, grease percentages, and yearling fleece weight. For the remaining mohair traits in which no statistically significant loci were observed using a quantitative variable, a case/control model was then applied using a threshold based on the median or quartile values to compare representatives demonstrating the greatest degree of phenotypic variation within the group. For traits that did not surpass an adjusted Bonferroni significance cutoff or an adjusted false discovery rate (FDR) significance cutoff of 0.05, we investigated significance using adaptive permutation for the model of best fit using PLINK v1.9 (Chang et al., 2015). Adaptive permutation evaluates the genomic dataset more quickly in that it discards SNPs which are not demonstrating association from further permutations, while continuing to analyze associated SNPs to the set threshold. Adaptive permutation output provides both the number of permutations achieved and corresponding *P*-value. Parameters used in the adaptive permutation testing included a minimum of five permutations performed but no more than 1,000,000 to determine significance using a confidence interval of 0.0001, alpha threshold of 0, intercept interval for pruning of 1, and slope interval of 0.001 for pruning (Che et al., 2014). Linkage disequilibrium (LD) structure and haplotype analysis was examined between associated markers using HAPLOVIEW v4.2 to assist in candidate gene identification (Barrett et al., 2005). Haplotypes blocks were defined using the algorithm from Gabriel et al. (2002). Putative candidate gene(s) within one million base pairs up or down stream or within LD blocks of significantly associated SNPs were identified based on the GCF-001704415.1(ARS1) assembly in Genome Data Viewer on National Center for Biotechnology Information (NCBI) (Bickhart et al., 2017).

## Results

### Statistical Analysis

Descriptive statistics of mean, standard deviation, minimum, and maximum for the seven fleece traits and yearling fleece weight are shown in **Table 2**. Covariate usage was determined using proc GLM in SAS for each GWAS (**Table 1**). No significant covariates were identified for kemp percentage or greasy fleece percentage. Sex was significant for diameter and true fiber percentage with a *P*-value < 0.0001, and for efficiency with a *P*-value < 0.05. Age was significant as a covariate for diameter and staple length with a *P*-value < 0.05, and for fleece weight with a *P*-value < 0.0001. Color was significant for diameter, true fiber, staple length and fleece weight with a *P* < 0.05. Least square means for yearling fleece weight in males was 392.4 ± 25.53, and 298.29 ± 18.69 in females (Tukey adjusted *P*-value < 0.0001). We normalized yearling fleece weight based on yearling body weight due to the positive correlation between these two traits.

**Table 2:** Descriptive statistics for the mohair traits evaluated.

Trait	Mean (*SD*)	Minimum	Maximum	Number^1^
Diameter	28.5 (± 3.8)	20.3	38.2	138
True fiber percentage	91.5 (± 9.6)	46	100	138
Percent kemp	4.9 (± 3.6)	1	22	115
Efficiency	25.6 (± 14.3)	4.8	68.2	138
Percent grease	2.1 (± 3.4)	0.02	38.7	138
Mature fleece weight	511.3 (± 240.1)	144	1260	127
Staple length	13.8 (± 2.9)	6.6	24.8	133
Yearling fleece weight	243.8 (± 117.1)	70	620	195

*^*1*^The number of individuals assessed.*

**FIGURE 2:**
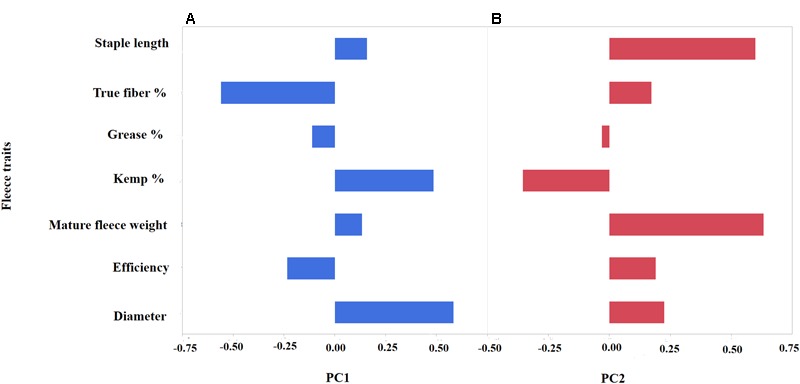
Principal Component Analysis (PCA) depicting the loading values (*x*-axis) of seven mohair traits (*y*-axis) for PC1 **(A)** and PC2 **(B)**. PC1 (blue) broadly describes “mohair quality” as indicated by the positive correlation between diameter and kemp percentage, while having negative correlation to true fiber percentage. PC2 (red) reflects fleece volume with a positive correlation between increased staple length and fleece weight.

To further investigate the level of variation of fiber traits in the Markhoz goats, we performed PCA. Principal components 1 and 2 cumulatively accounted for 56.1% of the total variance and were retained for further analysis. The specific fiber traits with absolute loading values greater than 0.4 were used to broadly describe the variation represented by each principal component. Principal component 1 (PC1) accounted for 29.9% of the total variance with the fiber traits for diameter and kemp percentage loading strongly on the positive side, while true fiber percentage loaded negatively (**Figure 2A**). PC1 broadly describes individual fiber quality with negatively scoring individuals having desirable mohair traits such as increased true fiber percentage and efficiency as the fiber diameter decreases and kemp percentage is reduced. Principal component 2 (26.2%) generally describes fiber volume, with fleece weight and staple length loading positively in contrast to kemp percentage loading negatively (**Figure 2B**). Animals with a positive PC2 score will have longer, thicker fibers supporting greater fleece weight. Coat color was significantly associated with PC2 (*P*-value < 0.05). Least square means for PC2 scores in black animals was 11.45 ± 2.03, in brown animals was 11.22 ± 2.05, and in white animals was 12.13 ± 2.11 (*P*-value < 0.0001). White animals, particularly in comparison to brown animals, demonstrated a higher value of PC2 (Tukey adjusted *P*-value < 0.05) which correlated to white animals producing a greater volume of fiber than colored animals.

### Coat Color Genome-Wide Associations

**FIGURE 3:**
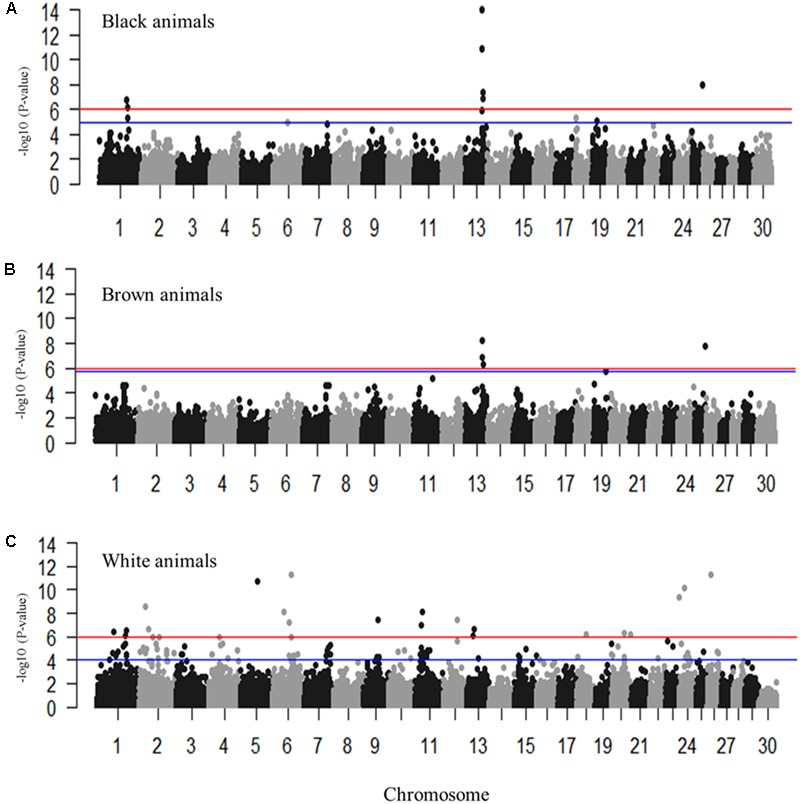
Manhattan plots for the coat colors of black, brown and white in Markhoz goats. **(A)** Black coat color contrasted to all non-black coat colors. **(B)** Brown coat color contrasted to all non-brown coat colors. **(C)** White coat color contrasted to all non-white coat colors. The red and blue horizontal lines indicate Bonferroni and FDR corrected *P*-values of 0.05, respectively.

Genome-wide association studies were performed for the coat colors of black, brown, and white utilizing an additive inheritance case-control model for each (**Figures 3A–C** and Supplementary Figures S1, S2). Utilizing the broader approach of comparing the coat color of interest (case) to all individuals not presenting that coat color (control), QTL were identified with proposed candidate genes (**Figures 3A–C**). Pairwise comparisons of each coat color also identified significantly associated markers despite the low sample sizes with results supporting the broader analysis (Supplementary Figure S2 and Supplementary Table S2). Significant regions associated with black coat color were located on chromosomes 1, 6, 13, 18, 19, and 25, with EMMAX *P*-values ranging from 1.20 × 10^-05^ to 9.62 × 10^-15^ (Supplementary Table S1). Loci for brown coat color were identified on chromosomes 13, 19, and 25, with EMMAX *P*-values ranging from 1.62 × 10^-06^ to 5.18 × 10^-09^ (Supplementary Table S1). The assessment of white coat color produced the greatest number of results with significant associations on 11 different chromosomes (passing both Bonferroni and FDR cutoffs) with EMMAX *P*-values ranging from 9.68 × 10^-05^ to 5.12 × 10^-12^ (Supplementary Table S1).

**FIGURE 4:**
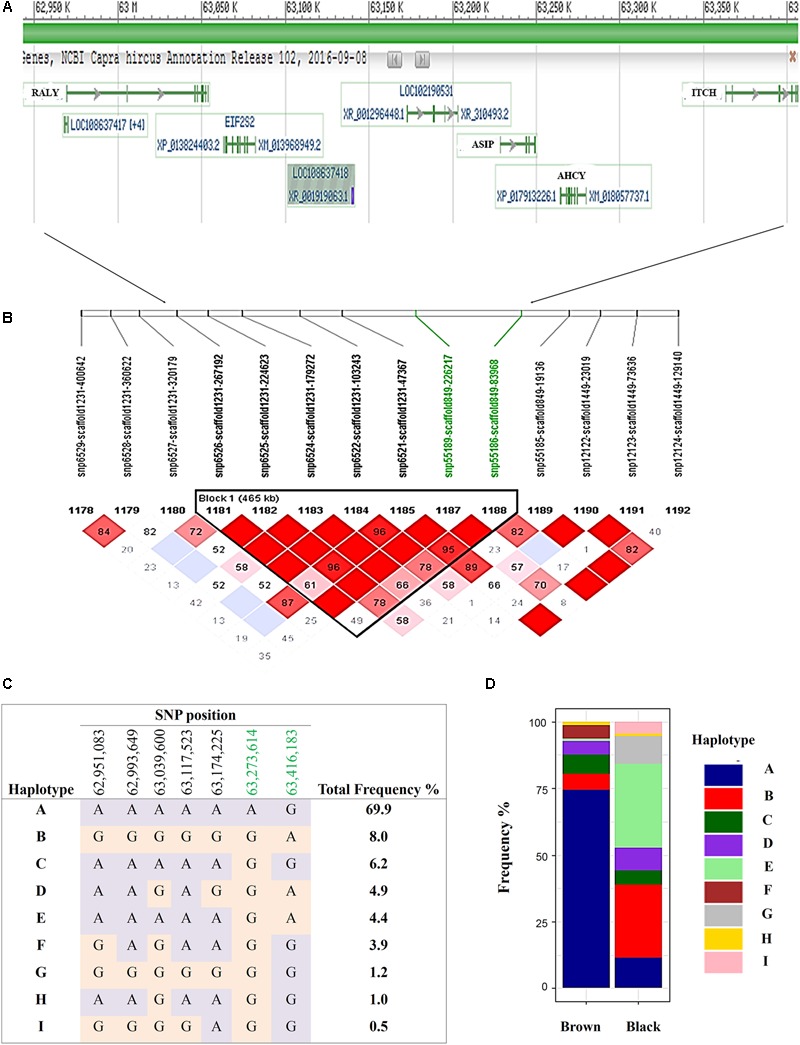
Haplotypes within a single 465 Kb LD block on chromosome 13 for brown and black coat color. **(A)** Genes incorporated in the **(B)** 465 Kb LD block **(C)** defined 9 haplotypes within the sampled Markhoz goat population and **(D)** the frequency of the haplotypes in black and brown goats. Gene track images are from NCBI Genome Data Viewer (https://www.ncbi.nlm.nih.gov/genome/gdv/browser/?acc=GCF_001704415.1&context=genome), accessed 10/11/2017).

The most significantly associated SNP for both the black and brown GWAS (**Figures 3A,B**) is located on chromosome 13 (snp55189-scaffold849-226217) within the Adenosylhomocysteinase (*AHCY*) gene. This SNP is within a 465 Kb block of LD encompassing *AHCY, ASIP*, and RALY heterogeneous nuclear ribonucleoprotein (*RALY*) genes, among others (**Figures 4A,B**). The second most associated SNP for black and third associated SNP for brown is also located on chromosome 13 (snp55186-scaffold849-83968) but within the itchy E3 ubiquitin protein ligase (*ITCH*) gene, also found in this same LD block (**Figures 4A,B**). The SNP on chromosome 25 falls within the sidekick cell adhesion molecule1 (*SDK1*) gene for both brown and black animals. The minor alleles and minor allele frequencies associated with these SNPs are presented in Supplementary Table S1. The pairwise comparison of black to brown supported these findings with variation in allele frequencies highlighting the same QTLs on chromosomes 13 and 25 (Supplementary Figure S2A and Supplementary Table S2). A new QTL was highlighted on chromosome 7 which narrowly missed the FDR threshold in the broader black comparison.

**FIGURE 5:**
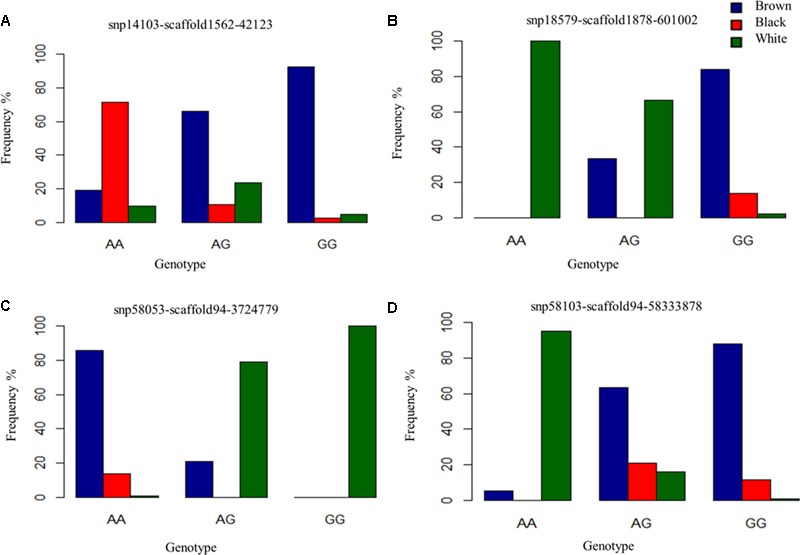
Genotype frequencies of prominently associated SNPs for each coat color. **(A)** Identifies a commonly associated SNP for black and brown coat color demonstrating variation in genotypic frequency. **(B–D)** Depict genotypic frequency distribution for three SNPs associated with white coat color.

Despite the common association of multiple SNPs for both brown and black coat color, genotypic frequencies and haplotype association reflect specific color descriptions. Nine haplotypes, incorporating seven SNPs, were identified within the 465 Kb LD block which included the above mentioned SNPs on chromosome 13 (**Figure 4C**). In total, five of the haplotypes were significantly associated with both black and brown coat color. Of the associated haplotypes, four of these were observed at a higher frequency in black animals while one was seen at a higher frequency in brown animals. A sixth haplotype was only associated and found at a higher frequency in brown animals. The percentage of each haplotype frequency found within black or brown coat color animals is depicted in **Figure 4D** and Supplementary Table S3. Genotypic frequencies for the individually associated SNP on chromosome 25 similarly reflects coat color variation between black and brown (**Figure 5A**).

For white animals, significant SNPs were detected on chromosomes 1, 2, 5, 6, 11, 12, 13, 18, 20, 26, and 24 with EMMAX *P*-values ranging from 9.68 × 10^-05^ to 5.12 × 10^-12^ (Supplementary Table S1). The most associated SNP (snp18579-scaffold1878-601002) is located on chromosome 26 (**Figure 3C**). However, the second associated SNP (snp58053-scaffold94-3724779) is on chromosome 6, relatively close (15,450 bp upstream) to the RAS like family 11 member B (*RASL11B*) gene but potentially more importantly, it is 1.6 Mb from the *KIT* gene (Supplementary Table S1). Association to the *KIT* gene is also supported by the association of snp58103-scaffold94-5833878, which is only 370 Kb downstream (Supplementary Table S1). Genotypic frequencies for these three SNPs are presented in **Figures 5B–D**. Pairwise comparison of white to black and white to brown (Supplementary Figures S2B,C, respectively) largely reflect the multiple signals found in the broader analysis of white coat color. This is particularly evident in the comparison of the brown to white individuals. However, the comparison of black to white individuals identified many new SNPs primarily on the same chromosomes and near the same regions. Novel QTL were identified on chromosomes 8 and 28 when comparing black to white individuals but yielded no candidate genes whereas the broader white comparison showed unique QTL on chromosomes 10, 11, 18, and 25.

### Mohair Trait Genome-Wide Association Studies

We performed eight GWASs for the mohair traits. Only GWAS for true fiber, efficiency, grease percentage and yearling fleece weight could surpass our Bonferroni or FDR corrected *P*-value of less than 0.05 (Supplementary Table S1 and Supplementary Figure S1). GWAS for PC1, PC2, diameter, staple length and mature fleece weight did not surpass the Bonferroni or FDR cutoff, but following one million permutations, candidate regions were identified for each trait (Supplementary Table S1 and Supplementary Figure S1).

**FIGURE 6:**
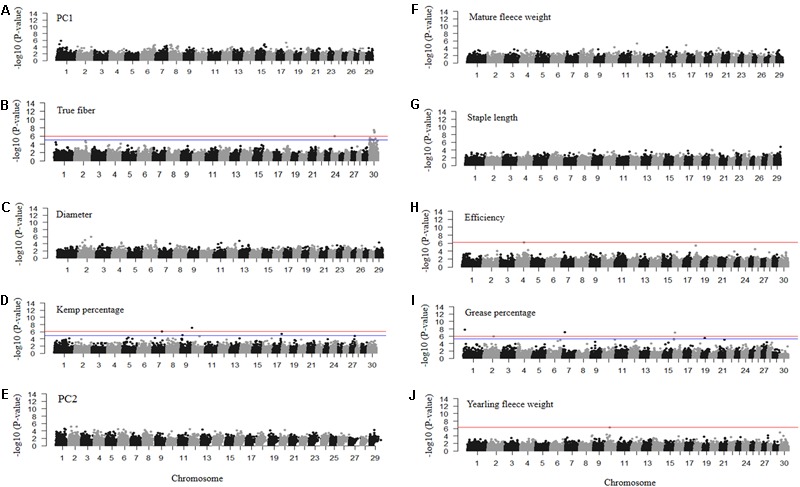
Manhattan plots of –log10 (*P*-values) for association of SNP loci for mohair traits **(A)** PC1 reflecting fiber quality, **(B)** true fiber percentage, **(C)** fiber diameter, **(D)** kemp percentage, **(E)** PC2 reflecting fiber volume, **(F)** mature fleece weight, **(G)** staple length, **(H)** efficiency, **(I)** grease percentage, and **(J)** yearling fleece weight. Traits with red and blue horizontal lines indicate Bonferroni and FDR corrected *P*-values of 0.05, respectively. Those with only a red horizontal line indicate *P*-values thresholds were the same for both Bonferroni and FDR. Those without horizontal lines reflect GWAS run using permutation testing.

The GWAS for PC1 representing fiber quality identified nine loci on chromosomes 1, 6, 8, 15, and 18 with permutated *P*-values ranging from 3.18 × 10^-05^ to 1.53 × 10^-06^ (**Figure 6A** and Supplementary Table S1). SNPs related to true fiber percentage were identified on chromosome 24 and X with the EMMAX *P*-values ranging from 8.78 × 10^-6^ to 3.55 × 10^-8^ (**Figure 6B**). After one million permutations, four SNPs associated to fiber diameter (**Figure 6C**) were identified on chromosomes 13 and 27 passing one million permutations and additional SNPs on chromosome 1 and 6 reaching 999,991 and 826,000 permutations with the *P*-value from 4.36 × 10^-5^ to 1.00 × 10^-6^ (**Figure 6C**), respectively. SNPs on chromosomes 6 and 13 were located within the secreted protein acidic and cysteine rich (*SPARC*) and solute carrier family 24 member 3 (*SLC24A3*) genes (Supplementary Table S1). There were only two significantly associated SNPs on chromosome 7 and 9 for kemp percentage with EMMAX *P*-values of 7.41 × 10^-07^ and 7.83 × 10^-08^ *P*-values, respectively, (**Figure 6D**).

Permutation testing of PC2 scores reflecting fiber volume identified six SNPs that passed one million permutations on chromosomes 1, 2, 6, and 12 with permutated *P*-values ranging from 3.79 × 10^-05^ to 6.00 × 10^-06^ (**Figure 6E**). The SNPs on chromosome 2 fell within the coiled-coil domain containing 148 (*CCDC148*) gene. SNPs for mature fleece weight, measured at the sampling age of the goats (1–8 years old), were unable to reach one million permutations, however, a SNP on chromosome 16 reached 734,732 permutations (**Figure 6F**) and is located within the uncharacterized *LOC102169208* gene (Supplementary Table S1). Staple length produced a SNP on chromosome 29 with a permutated *P*-value of 1.58 × 10^-05^ after reaching 886,838 permutations (**Figure 6G**) that fell within the potassium voltage-gated channel subfamily Q member 1 (*KCNQ1*) gene. The GWAS for efficiency only identified one significantly associated SNP on chromosome 4 with an EMMAX *P*-value of 6.38 × 10^-07^ (**Figure 6H**) within the inner mitochondrial membrane peptidase subunit2 (*IMMP2L*) gene. Grease percentage had multiple significantly associated SNPs on chromosomes 1, 2, 7, 16, and 19 with EMMAX *P*-values ranging from 8.36 × 10^-06^ to 1.72 × 10^-08^ (**Figure 6I** and Supplementary Table S1). The GWAS for yearling fleece weight identified one associated SNP on chromosome 10 with EMMAX *P*-value 5.21 × 10^-07^ (**Figure 6J**) within the sorbitol dehydrogenase (*SORD*) gene (Supplementary Table S1).

## Discussion

Markhoz goats are one of the few mohair-producing breeds and, due to their important cultural and economic roles, have unique coat color diversity with selection toward the brown coat color as opposed to the traditionally white Angora mohair. Thus, it would be extremely valuable to identify genetic variants and the underlying genes related to coat color and mohair traits. Recently, the release of an improved goat genome assembly (Bickhart et al., 2017) and caprine 50K SNP beadchip have offered more opportunities to examine the genetics of economically important traits in the goat (Tosser-Klopp et al., 2014). Here, we have identified multiple QTL and several putative candidate genes associated with coat color and mohair traits through genome-wide association that warrant further investigation for causative effect and potential use for genomic selection.

### Coat Color Loci

Association mapping for the black and brown coat colors separately identified a major locus on chromosome 13 (**Figures 3A,B**). Based on LD, the target region expanded to include the *AHCY, ASIP, RALY*, and *ITCH* genes (**Figures 4A,B**). The *ASIP* gene is well known for its role in coat color across several species and for its epistatic interaction with *MC1R* gene (Graham et al., 1997). Genetic variations within *ASIP* have been associated with coat color variation in other goat breeds such as the Saanen breed. Fontanesi et al. (2009b) reported that the dominant A^Wt^ allele can lead to white color in Saanen goats while a comparison of eight different goat breeds identified numerous missense mutations in the *ASIP* gene and a copy number variant (CNV) in *ASIP* and *AHCY* genes (Fontanesi et al., 2009b). The CNV is suggested to be responsible for introducing the A^Wt^ allele into both the Girgentana and Saanen goat breeds in a similar manner as observed in sheep previously (Norris and Whan, 2008). A GWAS for pink and pink necks in Saanen goats identified a QTL near the *ASIP* gene as well (Martin P.M. et al., 2016). Given the dominant effect of the *ASIP* allele for light coat color in Saanen goats and sheep, we suspect the recessive *ASIP* allele may contribute to the darker coat color of the Markhoz goats, but further testing is needed.

While *ASIP* is likely the major contributor to brown and black coat color in the Markhoz goat, the strong LD within the region identified specific haplotypes for brown vs. black coat color and suggests the *AHCY, RALY*, and *ITCH* genes could play regulatory roles in coat color. Significantly associated SNPs fell within each of these genes. Norris and Whan (2008) reported that a duplication containing the coding regions of *ASIP* and *AHCY*, and the *ITCH* promoter site is responsible for white coat color in sheep. The *ITCH* gene plays a role in apoptosis in melanoma cells (Yang et al., 2010). Melanoma is also a well-known skin cancer related to skin pigmentation. Individuals with fairer skin color, lighter hair and eye color are at higher risk for melanoma (Bradford, 2009) which supports a potential relationship between the *ITCH* gene and coat color, potentially contributing to the brown color variation observed. In horses, a duplication of the syntaxin 17 (*STX17*) gene is responsible for the gray coat color and melanoma, with increased susceptibility to melanoma in horses homozygous for the recessive *ASIP* allele (Rosengren Pielberg et al., 2008), indicating it is plausible for the *ITCH* gene to have similar pleiotropic effects. While further study is required to unravel the molecular interactions of the region in the Markhoz goats, the haplotypes we have identified for coat color (**Figures 4C,D**) could be valuable today in the genetic selection of black and brown animals.

A prior study of brown coat color in goats identified a non-synonymous variant in the *TYRP1* gene region on chromosome 8 (Becker et al., 2015). However, we were unable to identify this region within our own sample population. We did identify a SNP within the *RALY* gene that is present only in our black animals. The lethal yellow Ay allele of *ASIP* is known to disrupt the structure and expression of the *RALY* gene (Michaud et al., 1993). The *RALY* gene has also been associated with the saddle tan phenotype in the black and tan Basset hounds and Pembroke welsh corgis (Dreger et al., 2013). Therefore, we suspect that *RALY* together with *ASIP* gene could have a potential role in black coat color.

Association mapping for white coat color identified 98 significantly associated SNPs spanning 11 chromosomes which was substantially greater than results for either black or brown coat color. Retrospective analysis of the dataset showed that all 26 white animals also had wattles (Supplementary Figure S3). Wattles are a hair-covered appendage consisting of skin, blood vessels, muscle and core cartilage with an unclear biological function (Imagawa et al., 1994). None of our black or brown animals had wattles, therefore we cannot differentiate if our results are associated with a white coat color or with the presence of wattles. We suspect we have captured regions associated with both traits as genes within the regions have roles in keratinocyte differentiation, tissue morphogenesis, and coat color.

*KIT* and platelet derived growth factor receptor alpha (*PDGFRA*), both on chromosome 6, are the most promising genes we detected for white coat color (**Figure 3C**). To date, no study has associated the *KIT* gene with coat color in goats despite several studies in other species identifying *KIT*’s role in coat color (Marklund et al., 1998; Pielberg et al., 2002; Fontanesi et al., 2010a,b, 2014; Wong et al., 2013; David et al., 2014; Yan et al., 2014; Holl et al., 2017). In pigs, the *PDGFRA* gene is shown to be tightly associated with the dominant white coat color (Johansson et al., 1992; Johansson Moller et al., 1996). Within the same region as *KIT* and *PDGFRA*, is the *KDR* gene, well-known for playing a role in angiogenesis, vascular development, and hematopoiesis regulation (Risau, 1997; Gogat et al., 2004). The gene complex of *KDR, KIT*, and *PDGFRA* has been associated with the reddening coat color pattern in Angus cattle (Hanna et al., 2014). While *KDR* has been identified as a putative candidate for coat color in the cattle study, we suspect *KDR* is more likely contributing to the wattle vascular development in our goats based on its known role in angiogenesis. The fact that these three genes, including *KIT* and *PDGFRA* which are likely influencing white coat color and *KDR* which might contribute to wattle development, are in the same region could explain why wattles are only present in the white goats within our dataset.

Additional candidate genes functionally related to pigmentation, hair growth, and keratinocyte differentiation have a less obvious influence on white coat color and suggest either a complex regulation of the trait or are instead related to the development of wattles or increased fiber volume. Indeed, PC2 was directly linked to white animals which produced more fiber. This further complicates the interpretation of genetic signatures associated with white animals but provides some insight as to why the GWAS for white produced so many QTL.

The GWAS for white coat color and PC2 independently highlighted overlapping QTL on chromosome 2 for which the midpoints of the respective QTL were 312 Kb apart. Within this region were the *MREG* gene, which regulates melanosome transfer for which inhibition results in skin lightening (Wu et al., 2012), and the Abca12 ATP-binding cassette sub-family A (ABC1), member 12 (*ABCA12*) gene which regulates keratinocyte differentiation and epidermal lipid transportation (Akiyama, 2014). The fibronectin 1 (*FN1*) gene, which regulates tissue morphogenesis (Foolen et al., 2016), is also in this region but seems a more likely candidate for wattle development.

The following genes were highlighted in QTL for white coat color and retrospectively, wattles. The RAB11 family interacting protein 2 (*RAB11FIP2*) gene is thought to suppress the internalization of epidermal growth factor receptors (*EGFR*) (Cullis et al., 2002) which activate hair growth in both the mouse and human (Moore et al., 1981; Mak and Chan, 2003) while the Receptor type K (*PTPRK*) gene can be regulated by TGF-β pathway which decreases the *EGFR* activation in human primary keratinocytes (Xu et al., 2015). The nuclear factor of activated T cells 1 (*NFACTC1*) gene plays a role in skin tumorigenesis regulation via DMBA metabolism in which the loss of expression of this gene will decrease the skin tumorigenesis (Goldstein et al., 2015). The glutaredoxin and cysteine rich domain containing 1 (*GRXCR1*) gene is known to influence hair cell development with protein expression in the sensory epithelia in the inner ear. Mutations in the *GRXCR1* have been linked to hearing loss in both mice and humans (Odeh et al., 2010).

When focusing solely on candidate genes plausible for wattle development, we identified the previously mentioned *FN1*, as well as the sarcoglycan gamma (*SGCG*), and iroquois homeobox 2 (*IRX2*). Mutations in *SGCG* have been associated with muscle degradation (El Kerch et al., 2014) and *IRX2* plays a role in digit formation (Zulch et al., 2001). Thus, we hypothesized that these genes may influence wattle development due to their roles in tissue morphogenesis, muscle, and digit development, respectively. Our data did not reveal a QTL in the *FMN1*/*GREM1* region on chromosome 10 which was previously associated with wattle formation in a genome-wide analysis of nine Swiss goat breeds (Reber et al., 2015). Breed variation or our confounding overlap of white coat color and fiber volume traits may have influenced the different results. In general, further studies are needed to decipher the roles of these genes for which their functional annotation suggests potential roles in coat color, wattle formation, and/or fiber volume.

### Mohair Traits

Two different strategies were applied for mapping mohair traits in our goat population. First, PCA was used to group overlapping mohair qualities correlating to broader characteristics such as fiber quality and volume, which were then mapped using the resulting principal components 1 and 2, respectively. Second, we mapped each individual fiber trait. This mapping strategy was planned for three reasons: (1) to identify QTL and candidate genes related to broader characteristics of mohair quality and quantity which are economically important, (2) to compare the results from the PCA GWAS to individual trait mapping to look for potential overlapping regions, and (3) identify regions driving the regulation of specific mohair traits.

Principal component 1 described overall fiber quality, with true fiber scores being negatively correlated to increased kemp and larger diameter. Permutation mapping of PC1 identified a region on chromosome 1 near the POU class 1 homeobox 1(*POU1F1*) gene. This gene is known to play an important role in wool production in sheep and in greasy fiber percentage and staple length in cashmere goats (Lan et al., 2009; Zeng et al., 2011; Sun et al., 2013). Additionally, other studies have demonstrated that *POU1F1* has some effect on growth and milk production in other mammals (Mura et al., 2012; Ozmen et al., 2014; Sadeghi et al., 2014). This is the first study to apply PCA to fiber traits for a broader perspective of the genetic regulation of overall mohair quality and quantity.

Association mapping for the individual traits of true fiber percentage and kemp percentage did not identify genes with obvious roles in hair or fiber characteristics. However, association studies on both PC1 and fiber diameter highlighted the same region on chromosome 1 close to the *POU1F1* gene described above. With PC2, we were able to describe overall fleece volume related to fleece weight and staple length. Candidate genes such as *MREG* and *ABCA12* were previously described as they overlapped with white coat color QTL. *ABCA12* has a more plausible relationship with fiber volume as it regulates keratinocyte differentiation and epidermal lipid transportation. Laminin subunit beta 3 (*LAMB3*) and hydroxysteroid 11-beta dehydrogenase 1 (*HSD11B1*) genes, which influence hair morphogenesis and dermatitis in both mice and humans, respectively, were identified as the most likely candidates for influencing mature fleece weight within the associated QTL (Imanishi et al., 2014; Terao et al., 2016). While the GWAS for fiber efficiency highlighted the novel gene of *IMMP2L*, previously unassociated with fiber traits, the association mapping for greasy fleece percentage produced more intriguing results. This included the adhesion G protein-coupled receptor V1 (*ADGRV1*) gene which has a role in the development of auditory hair bundles in mice and is related to Usher syndrome, a highly heritable disease which consists of various symptoms including hearing loss and vision impairment (McGee et al., 2006; Kahrizi et al., 2014; Yang et al., 2016). It would be of interest to explore the role of auditory hairs, which collect wax within the ear canal, and the incidence of deafness among the goats. Ironically, both *ADGRV1* and *GRXCR1* are associated with auditory hair development and mutations are linked to hearing loss. Another gene includes the histamine *N*-methyltransferase (*HNMT*) gene, which has a link to skin lesions in mice (Furukawa et al., 2009). As these genes are involved in hair and skin disorders, they might influence fiber development and fiber traits both directly and indirectly via different pathways.

Lastly, the GWAS for yearling fleece weight, which is related to a finer mohair yield due to the younger age of the animals, was conducted. As the goats increase in age, the mohair fiber becomes more coarse as the fiber diameter increases (Mohair South Africa, 2017). The *SORD*, dual oxidase 1 (*DUOX1*), dual oxidase maturation factor 2 (*DUOXA2*), dual oxidase 2 (*DUOX2), dual oxidase maturation factor 1(DUOXA1)*, and arginine-glycine aminotransferase (*GATM*) genes on chromosome 10 reside in the associated QTL for yearling fleece weight. The *SORD* gene is regulated by androgens and expressed in epithelial cells (Szabo et al., 2010). Coincidently, our data showed a positive correlation between increased yearling fleece weight and the male sex which we hypothesize may be due to differential regulation of the *SORD* gene. Studies in humans have reported that *DUOX1* plays a role in the expression levels of normal keratinocytes (Choi et al., 2014). As keratinocytes produce keratin, the main protein for hair, nail, and skin synthesis, we hypothesize that this gene may be associated with additional fiber development. Although not related to fiber, a deficiency in the *GATM* gene is related to an autosomal-recessive disorder with varying symptoms including myopathy (Choe et al., 2013; Stockler-Ipsiroglu et al., 2015). The analysis of yearling fleece weight was normalized as it was positively correlated with yearly weight likely reflecting muscle mass.

In all, many of our QTL differed from previous mapping studies for fiber related traits (Cano et al., 2007, 2009; Mohammad Abadi et al., 2009; Visser et al., 2011; Roldan et al., 2014). Disagreement in our findings compared to these studies is likely due to breed differences as well as marker placement and density used in the analysis. Fine mapping and expression studies of some of the unique fiber quality related genes such as *POU1F1. ADGRV1, MREG, LAMB3*, and *HSD11B1* may lead to new insight toward biological pathways influencing hair development and growth. In contrast, our QTL related to coat color highlighted candidate genes extensively documented for influencing color patterns in goats as well as a variety of other species. Future fine mapping of the identified regions, especially *ASIP, RALY, KIT*, and *PDGFRA* genes is needed to identify causal mutations or other structural phenomenon such as CNVs that are contributing to coat color of the Markhoz breed. Unexpectedly, we were also able to highlight genes potentially influencing the presence of wattles, which currently remain a biological mystery.

Despite advances in the genome assembly and tool development in several species over the last 10 years, there have been few genome scale studies for traits considered economically important in goats, particularly in Angora and Cashmere goats. This is the first genetic study to identify regions associated with coat color and fiber traits in the Markhoz breed as well as potential SNPs and haplotypes for genetic selection of coat color. The relatively small cohort of goats investigated was a limiting factor yet provided biological insight for these traits and a foundation to further genomic research on coat color and mohair traits in goats.

## Data Availability

All SNP genotype data are available at the publically assessable Zenodo repository (https://zenodo.org/record/1198730#.Wq lmbcPwZdg).

## Author Contributions

All authors were engaged in the development of the overall research plan and assisted with research advisement. AN-G was the lead researcher performing data collection, statistical and genomic analysis, result interpretation, and drafting of the manuscript. AR provided assistance with sample and data collection of the Markhoz goats. HM-Y and SM-A provided primary advisement during sample collection and laboratory support for DNA extraction. ES assisted with the genomic methodology, data analysis, result interpretation, and initial manuscript draft. HH managed the genotyping and directed the genomic data analysis, result interpretation, and was the primary editor of the manuscript.

## Conflict of Interest Statement

The authors declare that the research was conducted in the absence of any commercial or financial relationships that could be construed as a potential conflict of interest.
