# Supplementary figures and images for: Genome-Wide Association Studies Identify Candidate Genes for Coat Color and Mohair Traits in the Iranian Markhoz Goat
Source: Front Genet. 2018 Apr 4;9:105. doi: 10.3389/fgene.2018.00105 (PMC5893768; doi:10.3389/fgene.2018.00105)

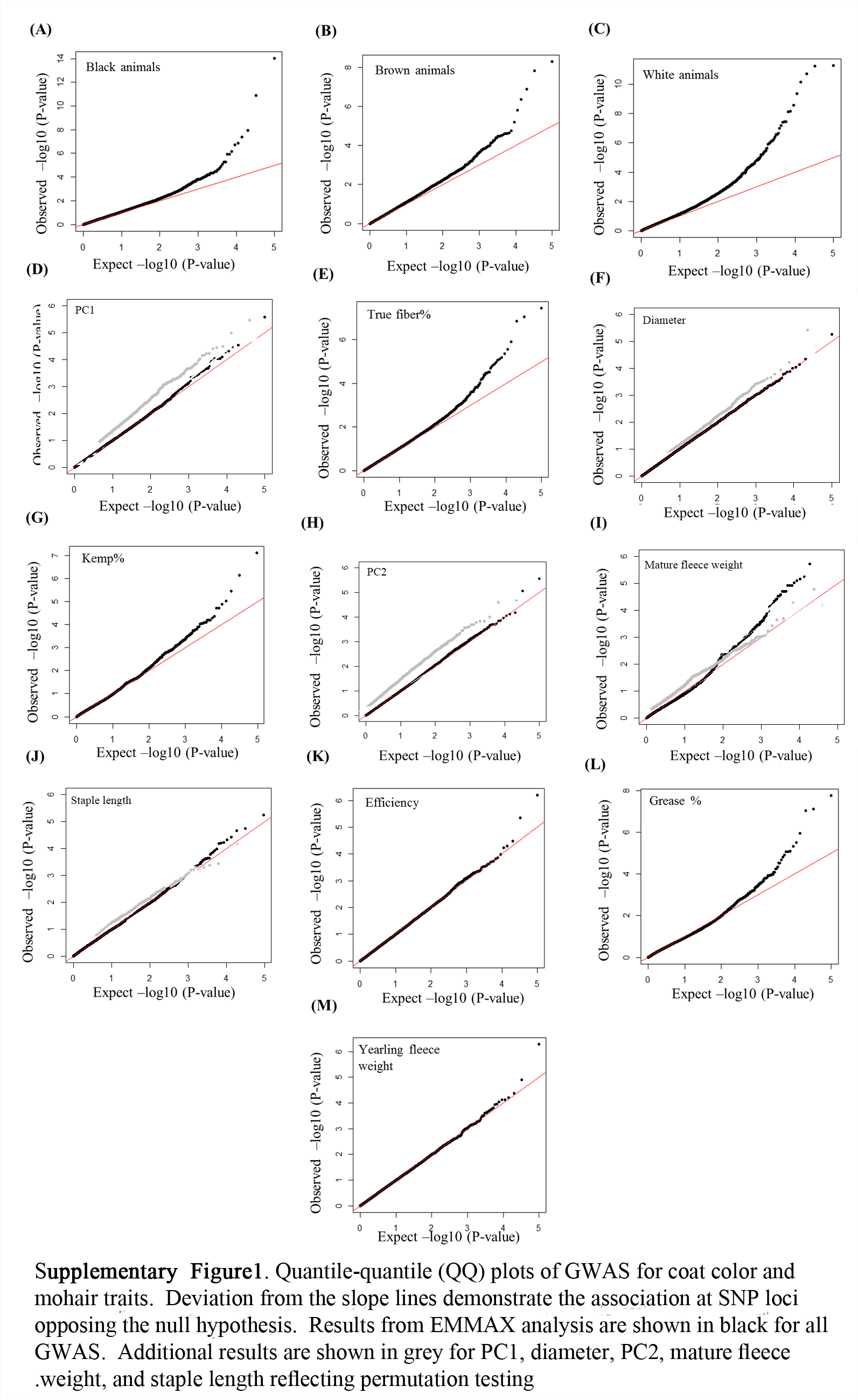

Supplement: Supplementary file 4 [file Image_1.TIF]

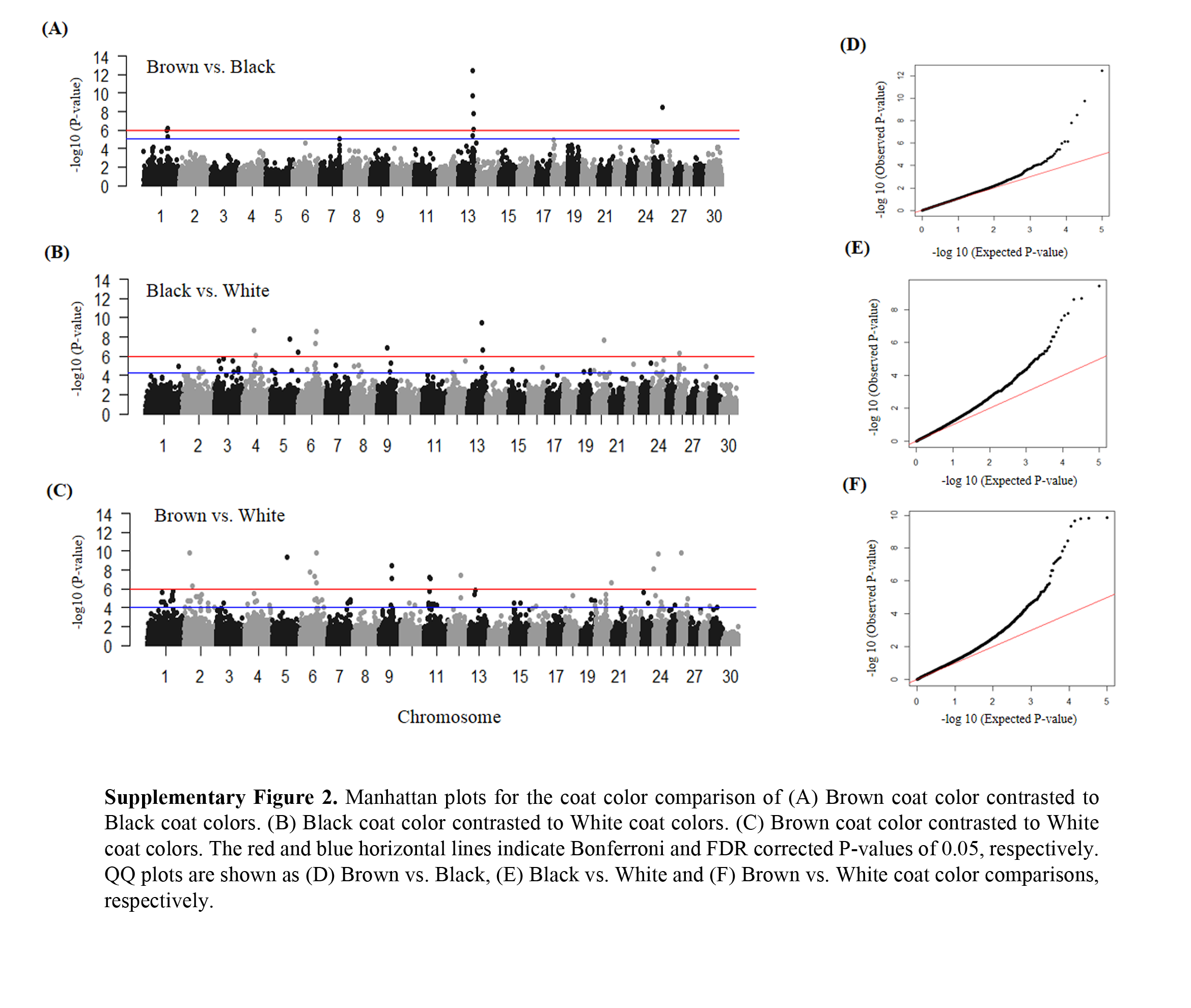

Supplement: Supplementary file 5 [file Image_2.tif]

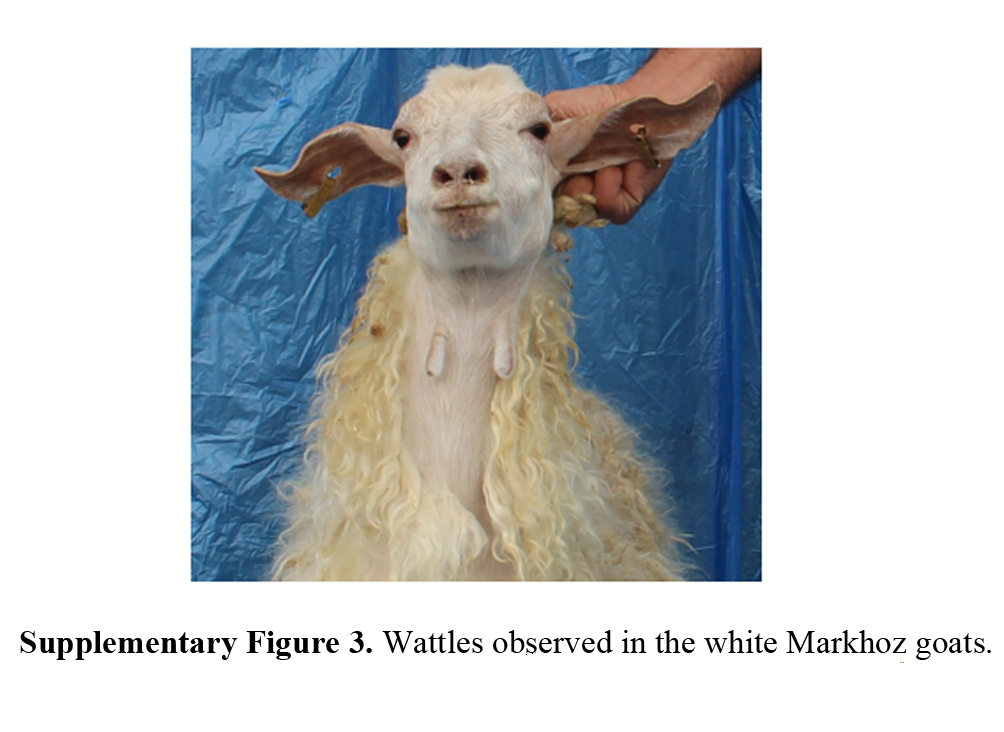

Supplement: Supplementary file 6 [file Image_3.tif]
